# Symptomatic gastric submucosal lipoma presenting with melena and iron-deficiency anemia in a 71-year-old male: a rare case report

**DOI:** 10.1097/RC9.0000000000000694

**Published:** 2026-07-15

**Authors:** Negin Haghshenas, Mehran Hatabi, Arash Dadvand, Reza Pezeshki, Mirsalim Seyedsadeghi, Atabak Sedigh-Namin

**Affiliations:** a Anesthesiology Department, Ardabil University of Medical Sciences, Ardabil, Iran; b Department of Surgery, School of Medicine, Ardabil University of Medical Sciences, Ardabil, Iran; c Students Research Committee, School of Medicine, Ardabil University of Medical Sciences, Ardabil, Iran; d Department of Anatomical Sciences and Pathology, School of Medicine, Imam Reza Hospital, Ardabil University of Medical Sciences, Ardabil, Iran

**Keywords:** gastric lipoma, gastrointestinal bleeding, melena, submucosal tumor

## Abstract

**Introduction and importance:**

Gastric lipomas are rare, benign mesenchymal tumors composed of mature adipose tissue, accounting for less than 3% of all benign gastric neoplasms. They are typically asymptomatic but can occasionally present with gastrointestinal bleeding or obstruction when large. Due to their submucosal origin and nonspecific clinical features, preoperative diagnosis can be challenging.

**Case presentation:**

A 71-year-old male presented with fatigue, melena, and hypotension. Laboratory tests revealed iron deficiency anemia (hemoglobin 7.9 g/dL) and thrombocytopenia. Upper GI endoscopy showed a submucosal lesion of size 4 × 5 cm in the distal gastric body with a central depression and visible vessel, suggestive of GIST, but a biopsy was not performed due to the submucosal location and high suspicion of a mesenchymal tumor. CT confirmed a well-circumscribed submucosal mass. The patient underwent distal gastrectomy with gastrojejunostomy. Histopathology revealed a submucosal lipoma with tumor-free margins.

**Clinical discussion:**

Although gastric lipomas are usually asymptomatic, larger lesions can lead to mucosal ulceration and bleeding. In such cases, surgical resection remains the treatment of choice, providing both diagnostic confirmation and complete cure. Awareness of this rare cause of upper GI bleeding can help prevent misdiagnosis as malignant tumors, such as GIST.

**Conclusion:**

Gastric lipoma, while rare, should be considered in elderly patients presenting with unexplained GI bleeding or anemia. Complete surgical excision offers an excellent prognosis.

## Introduction

Gastric lipomas (GLs) are uncommon benign tumors composed of mature adipose tissue, accounting for 1–3% of all gastric tumors and approximately 2–3% of benign gastric neoplasms^[^1–3^]^. They most commonly arise in the submucosal layer, with a predilection for the gastric antrum or pyloric region, and are typically discovered incidentally during imaging or endoscopic procedures^[^4–6^]^. While small lipomas are generally asymptomatic, larger lesions may present with clinical symptoms, including gastrointestinal (GI) bleeding, abdominal pain, obstruction, or, rarely, gastroduodenal intussusception^[^7–9^]^.

HIGHLIGHTS

- Gastric lipomas are rare benign tumors, accounting for less than 3% of all benign gastric neoplasms.
- Large lesions (>4 cm) may present with gastrointestinal bleeding or anemia due to mucosal erosion or pressure necrosis.
- CT imaging is the most reliable diagnostic tool, showing a well-circumscribed, homogeneous, fat-density lesion.
- Surgical resection remains the treatment of choice for symptomatic or large gastric lipomas.
- Early recognition can prevent complications and ensure excellent postoperative outcomes.

Diagnosis can be challenging due to the submucosal origin and nonspecific presentation. Imaging modalities, such as computed tomography (CT) and endoscopy, play a key role in identifying these tumors, with CT being particularly useful for demonstrating the fatty composition of the lesion^[^10^]^. Although GLs are mostly detected in the fifth or sixth decade of life, no significant sex predilection has been observed^[^11^]^.

Management depends on tumor size and symptomatology. Small, asymptomatic lesions may be monitored without intervention, whereas larger or symptomatic lipomas often necessitate endoscopic or surgical removal to prevent complications and to differentiate them from other gastric masses, such as adenocarcinomas, lymphomas, or ulcerated lesions with mass effect^[^12,13^]^.

Here, we present a case of a 71-year-old man who presented with weakness and fatigue and was subsequently diagnosed with a GL, highlighting the clinical features, diagnostic work-up, and management of this rare entity.

This case report has been prepared in accordance with the SCARE 2025 criteria to ensure a structured and comprehensive presentation^[^14^]^.

## Case report

A 71-year-old male was admitted to our center with a 1-month history of progressive fatigue, generalized weakness, and low-grade fever. During the week prior to admission, he developed melena and symptomatic hypotension. One day before hospitalization, he experienced an episode of non-bloody vomiting. He denied nausea, anorexia, abdominal pain, or weight loss. On presentation, the patient appeared pale and fatigued. Vital signs were as follows: blood pressure 90/60 mmHg, heart rate 110 bpm, respiratory rate 18/min, oxygen saturation 94% on room air, and temperature 37.1 °C.

The patient had a 10-year history of simple hepatic and right renal cysts. Several years earlier, the right renal cyst had been aspirated under ultrasound guidance, followed by ethanol injection, which resulted in the complete resolution of the lesion. The hepatic cyst, however, persisted as a simple cyst in the right lobe of the liver, as also noted on the current contrast-enhanced CT scan.

In the days leading up to admission, he experienced episodes of tachycardia and dizziness. He first visited a cardiologist, and cardiac causes were ruled out (R/O cardiac disease). Due to persistent dizziness, he was referred to a neurologist, and a brain CT scan was normal. The underlying cause of these symptoms was later found to be occult GI bleeding, which had gone unrecognized until a general practitioner performed a digital rectal examination and detected melena, prompting referral to our hospital.

On presentation, the patient appeared pale and fatigued. Vital signs revealed hypotension, whereas cardiovascular and respiratory examinations were within normal limits. An abdominal examination showed no tenderness, distension, or organomegaly.

Initial laboratory investigations demonstrated iron deficiency anemia (hemoglobin 7.9 g/dL, ferritin 13 ng/mL), thrombocytopenia (platelet count 96 000/mm^3^), and elevated urea (54 mg/dL) with normal creatinine – findings consistent with upper GI blood loss (Table 1).

**Table 1:** Laboratory findings on admission demonstrating iron deficiency anemia (hemoglobin 7.9 g/dL, ferritin 13 ng/mL), thrombocytopenia (platelet count 96 000/mm^3^), and elevated urea (54 mg/dL) with normal creatinine. Reference ranges are provided for each parameter.

Category	Test	Result	Unit	Reference range
Hematology	WBC	4600	cu/mm	4000–10 000
	RBC	**2.94 L**	Mill/mm^3^	M: 4.5–6.3/F: 4.2–5.4
	Hb	**7.9 L**	g/dL	M: 14–18/F: 12–16
	Hct	**27.2 L**	%	M: 39–52/F: 36–46
	MCV	93	fL	80–96
	MCH	27	pg	26–32
	MCHC	29	g/dL	26–36
	Platelet	**96 000 L**	/mm^3^	150 000–450 000
	Poly (Neut)	74	%	–
	Lymph	18	%	–
	Mono	5	%	–
	Eos	3	%	–
Biochemistry	Urea	**54 H**	mg/dL	15–45
	Creatinine	0.97	mg/dL	0.5–1.4
	SGOT (AST)	20	IU/L	5–40
	SGPT (ALT)	14	IU/L	5–40
	Alk. P	188	IU/L	80–306
	Total bilirubin	0.85	mg/dL	0.2–1.2
	Direct bilirubin	0.35	mg/dL	0–0.4
	Serum Na	145	mEq/L	132–145
	Serum K	3.7	mEq/L	3.7–5.5
Iron studies	Serum iron	80	mcg/dL	M: 60–180/F: 40–155
	TIBC	354	mcg/dL	230–440
	Ferritin	**13 L**	ng/mL	M: 21.8–274.7/F: 4.6–204

Bold values indicate laboratory test results outside the normal reference range.

Despite the transfusion of three units of packed red blood cells, his hemoglobin level did not increase, as he continued to bleed during hospitalization. Consequently, a surgical consultation was requested.

Upper GI endoscopy revealed a submucosal lesion of size 4 × 5 cm located in the distal gastric body, extending toward the proximal antrum. The lesion exhibited a central depression with a visible vessel. Based on its endoscopic appearance, a preliminary diagnosis of GI stromal tumor (GIST) was suggested. Endoscopic biopsy was not attempted because the lesion was submucosal, and endoscopic forceps biopsy is typically nondiagnostic for such deep lesions. No endoscopic variceal ligation was performed, as there were no varices.

**Figure 1.**
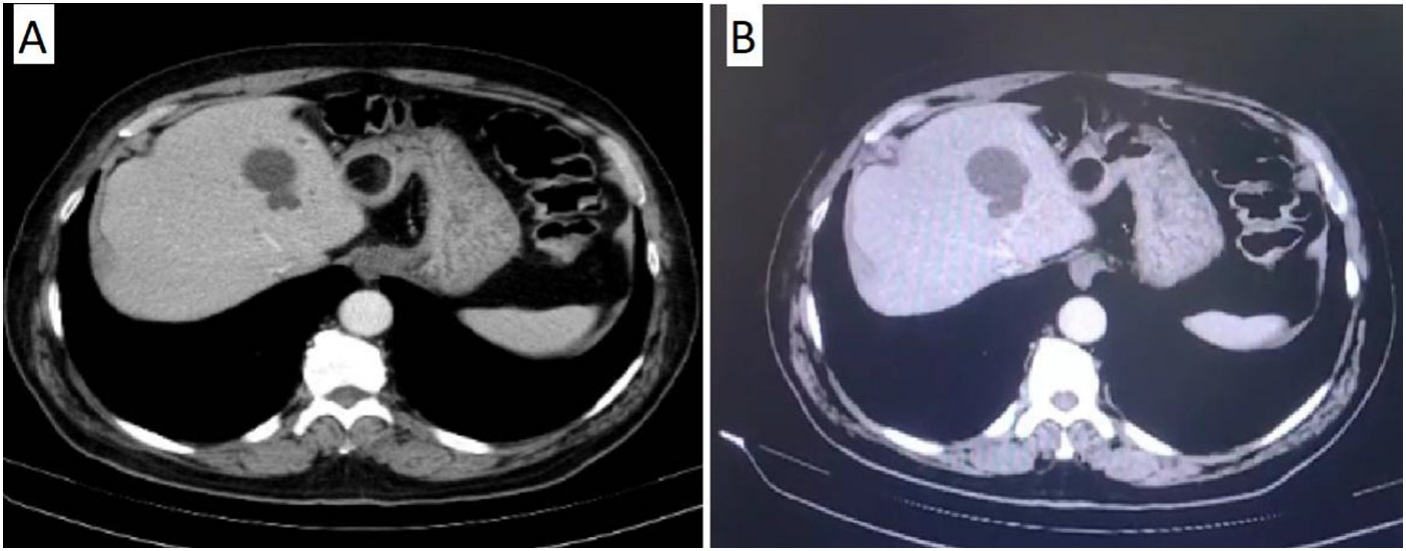
A contrast-enhanced abdominal CT scan showing a well-defined submucosal mass in the distal stomach, with no evidence of metastatic disease.

A contrast-enhanced CT scan of the abdomen confirmed the presence of a well-defined submucosal mass in the distal gastric region, without evidence of metastatic disease, and also demonstrated the previously mentioned simple hepatic cyst in the right lobe (Fig. 1).

**Figure 2.**
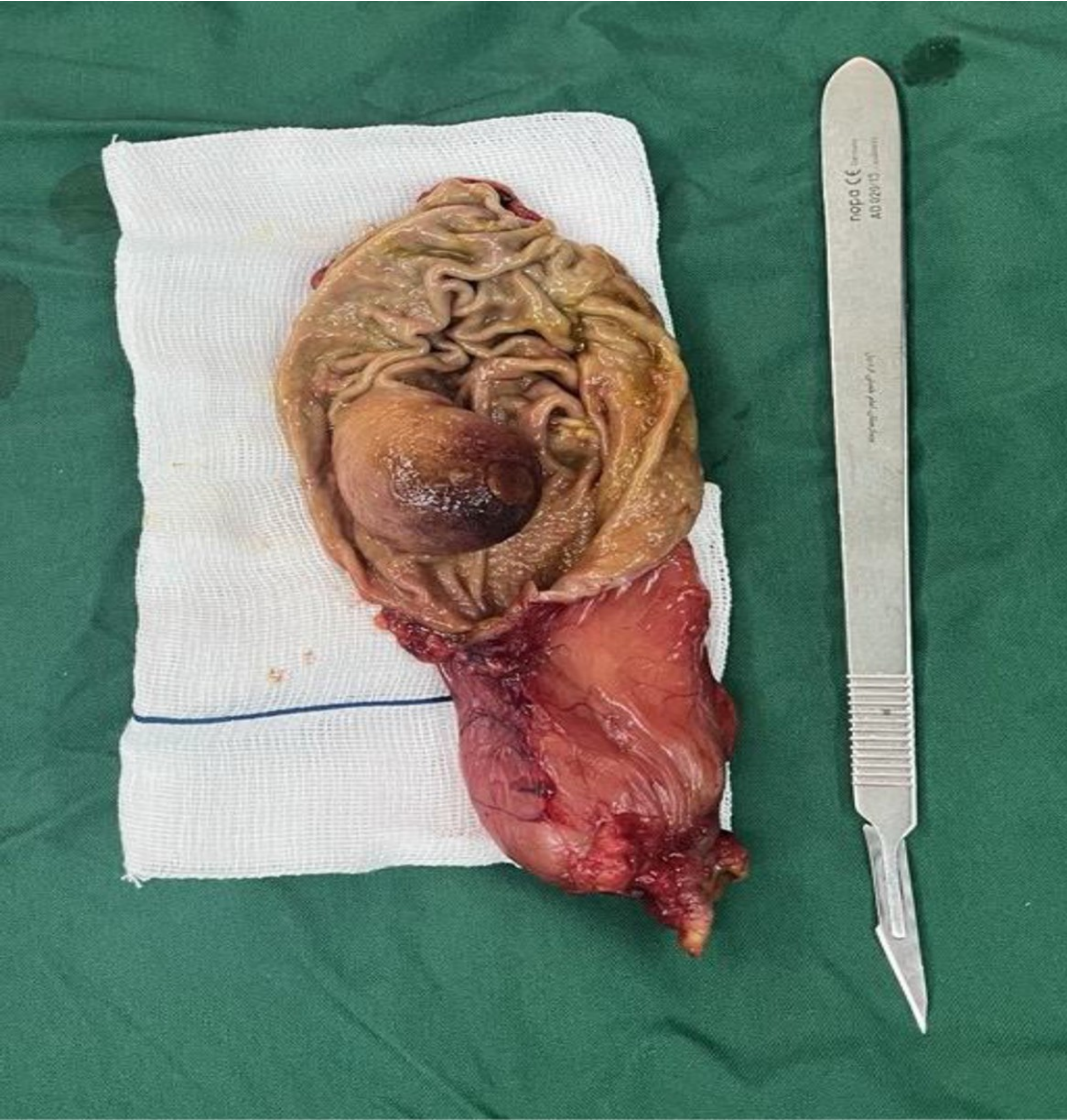
Gross specimen of the resected gastric mass, showing a well-encapsulated, lobulated lesion consistent with a submucosal tumor.

Laparoscopic approach was considered; however, due to the patient’s hemodynamic instability on admission (persistent hypotension despite transfusion) and the large tumor size (4 × 5 cm) with suspected deep invasion, an open exploratory laparotomy was preferred to ensure safe resection and hemostasis. Given the lesion’s size, symptomatic presentation, and potential for complications, the patient underwent exploratory laparotomy. Intraoperatively, a large, well-encapsulated mass was identified in the distal gastric wall. A distal gastrectomy with gastrojejunostomy was performed, and a drain was placed for postoperative management (Fig. 2).

**Figure 3.**
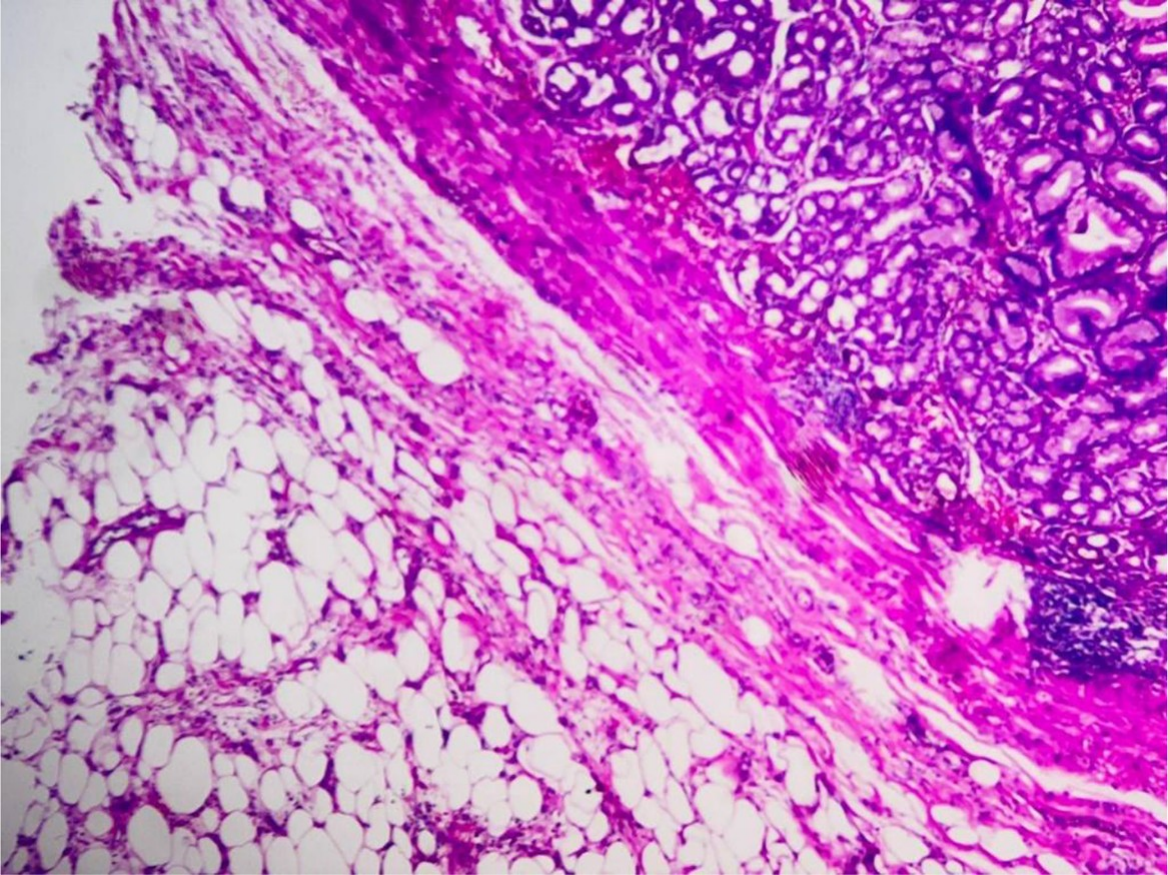
Histopathological image of the resected specimen, showing mature adipose tissue consistent with a submucosal lipoma (H&E stain).

Histopathological evaluation of the resected specimen revealed mature adipose tissue consistent with a submucosal lipoma. Both resection margins were free of tumor. The patient’s postoperative recovery was uneventful, and he was discharged in stable condition with a scheduled follow-up (Fig. 3).

At 6-month follow-up, the patient remained asymptomatic, with normalized hemoglobin (13.2 g/dL) and no evidence of recurrence on CT imaging.

## Discussion

GLs are rare benign mesenchymal tumors derived from mature adipose tissue, representing less than 3% of all benign gastric neoplasms and approximately 5% of GI lipomas^[^1,2^]^. They are most frequently located in the gastric antrum and typically arise from the submucosal layer, although subserosal forms have also been described^[^4,6^]^. The etiology of these lesions remains unclear, and their clinical presentation is largely determined by the size and location of the tumor.

Small GLs are usually asymptomatic and are discovered incidentally during endoscopy or imaging. In contrast, larger lesions (>4 cm) may present with GI bleeding, obstruction, or anemia due to mucosal ulceration or pressure necrosis^[^6,7^]^. In our patient, the lesion measured approximately 4 × 5 cm and manifested as melena, fatigue, and anemia, consistent with previous reports describing symptomatic cases associated with large GLs. Similar cases of large GLs presenting with melena have been reported by Ramdass *et al* (2013) and Yen *et al* (2018), both of whom also underwent surgical resection with favorable outcomes.

Endoscopically, GLs often appear as smooth, well-circumscribed yellowish masses covered by intact or eroded mucosa. Certain characteristic features – such as the “pillow,” “tenting,” and “naked fat” signs – can aid in their identification^[^15^]^. However, endoscopic biopsies are frequently non-diagnostic, as the lesion is submucosal in nature. Imaging techniques, such as CT, are therefore essential for diagnosis, typically revealing a well-defined, homogeneous, low-attenuation mass consistent with the fatty density^[^16^]^. In our case, CT imaging demonstrated a submucosal mass in the distal gastric region with no evidence of metastasis, strongly suggestive of a benign lipomatous lesion.

The main differential diagnoses for GLs include the GIST, liposarcoma, leiomyoma, and glomus tumor^[^11^]^. Distinguishing lipomas from these entities is critical, as management strategies differ significantly. Although GLs are benign and have no documented risk of malignant transformation, their clinical relevance arises when they cause complications such as bleeding, ulceration, or gastric outlet obstruction^[^17^]^.

Treatment decisions depend on the lesion’s size, symptoms, and potential for complications. Small, asymptomatic lipomas may be observed conservatively, whereas larger or symptomatic lesions typically require removal^[^18^]^. Both endoscopic and surgical approaches have been described. Endoscopic methods – including endoscopic mucosal resection and endoscopic submucosal dissection – are effective for smaller, accessible lesions (a-9, c-9). However, for large, deeply seated, or bleeding lesions, partial gastrectomy or wedge resection remains the preferred approach^[^19^]^.

In our case, given the lesion’s size, symptomatic presentation, and initial suspicion of GISTs, a distal gastrectomy with gastrojejunostomy was performed. Histopathological examination confirmed a submucosal lipoma with negative resection margins. The patient’s postoperative recovery was uneventful, and his clinical condition improved markedly.

This case underscores the importance of considering GLs as a differential diagnosis in elderly patients presenting with upper GI bleeding or unexplained anemia. Early recognition using imaging modalities and timely surgical management can lead to excellent outcomes, preventing unnecessary radical procedures and reducing morbidity.

## Conclusion

GLs, although rare, should be considered in the differential diagnosis of elderly patients presenting with unexplained upper GI bleeding or iron-deficiency anemia. CT imaging plays a key role in preoperative diagnosis by demonstrating the fatty nature of the lesion. Complete surgical excision, as performed in our patient, offers an excellent prognosis with no risk of recurrence when margins are negative. This case highlights the importance of recognizing benign GLs to avoid unnecessary radical surgery and ensure appropriate management.

## Data Availability

Due to the sensitive nature of the clinical data and patient confidentiality, the datasets generated and/or analyzed during the current study are not publicly available. De-identified data may be available from the corresponding author upon reasonable request and with the permission of the Ethics Committee of Ardabil University of Medical Sciences.
